# Lipidomic profiling of chorionic villi in the placentas of women with chronic venous disease

**DOI:** 10.7150/ijms.49236

**Published:** 2020-09-30

**Authors:** Miguel A Ortega, Miguel A Saez, Felipe Sainz, Oscar Fraile-Martínez, Sandra García-Gallego, Leonel Pekarek, Coral Bravo, Santiago Coca, Melchor Álvarez- Mon, Julia Buján, Natalio García-Honduvilla, Ángel Asúnsolo

**Affiliations:** 1 Department of Medicine and Medical Specialties, Faculty of Medicine and Health Sciences, University of Alcalá, Alcalá de Henares, Madrid, Spain.; 2 Ramón y Cajal Institute of Healthcare Research (IRYCIS), Madrid, Spain.; 3 University Center for the Defense of Madrid (CUD-ACD), 28047 Madrid, Spain.; 4 Pathological Anatomy Service, Central University Hospital of Defence-UAH Madrid, Spain.; 5 Department of Surgery, Medical and Social Sciences, Faculty of Medicine and Health Sciences, University of Alcalá, Alcalá de Henares, Madrid, Spain.; 6 Angiology and Vascular Surgery Service, Central University Hospital of Defence-UAH Madrid, Spain.; 7 Department of Organic and Inorganic Chemistry, and Research Institute in Chemistry “Andrés M. del Río” (IQAR), University of Alcalá, 28805 Madrid, Spain.; 8 Service of Gynecology and Obstetrics, Section of Fetal Maternal Medicine, Central University Hospital of Defence-UAH Madrid, Spain.; 9 Internal Medicine and Oncology Service Service, University Hospital Príncipe de Asturias, CIBEREHD, Alcalá de Henares, Madrid, Spain.; 10 Department of Epidemiology & Biostatistics, Graduate School of Public Health and Health Policy, University of New York, New York, NY, United States.

**Keywords:** Chronic venous disease, pregnancy, chorionic villi, triglycerides, sphingomyelins or non-esterified omega 9 fatty acids

## Abstract

**Background:** Chronic venous disease (CVD) is a prevalent lower limb venous pathology that especially affects women, who also show an increased risk of this disease during pregnancy. Studies have shown significant structural changes in the placentas of women with CVD and several markers of tissue damage have been also described.

**Patients and Methods:** To try to understand the different placental pathologies, research efforts have focused on examining metabolomic profiles as indicators of the repercussions of these vascular disorders. This study examines changes produced in the metabolomic profiles of chorionic villi in the placentas of women with CVD. In a study population of 12 pregnant women, 6 with and 6 without CVD, we compared through mass spectroscopy coupled to ultra-high performance liquid chromatography (UHPLC-MS), 240 metabolites in chorionic villus samples.

**Results:** This study is the first to detect in the placental villi of pregnant women with CVD, modifications in lysophosphatidylcholines and amino acids along with diminished levels of other lipids such as triglycerides, sphingomyelins, and non-esterified omega 9 fatty acids, suggesting a role of these abnormalities in the pathogenesis of CVD.

**Conclusions:** Our findings are a starting point for future studies designed to examine the impacts of CVD on maternal and fetal well-being.

## Introduction

Chronic venous disease (CVD) is a common condition in which venous return to the heart is impaired with clinical manifestations such as varicose veins 1, 2. Its prevalence has been estimated at around 30% 3. The most frequent cause of CVD is a situation of venous hypertension along with an inflammatory response 4. Risk factors for CVD include obesity, ageing, family history, occupation and pregnancy 5-7. During pregnancy it is estimated that some 1 in 3 women will develop this disease. Throughout pregnancy the great variety of changes that occur in a woman's body may promote the appearance of CVD. These changes include elevated levels of estrogens or progesterone that will induce a series of hemodynamic changes such as increased plasma volume or vein diameter along with impaired vein valve closure leading to reduced peripheral blood system resistance 8-10.

However, despite its high prevalence, the role of venous hypertension induced by CVD in the placenta has not been as well described as the role of preeclampsia, its analogous arterial system condition. Preeclampsia is a systemic disease of pregnancy that is life threatening for the mother and fetus characterized by an intense inflammatory response, endothelial damage, greater platelet aggregation and blood clotting cascade activation, among other factors 11. Several pathogenic mechanisms that affect the placenta in this disease have been described such as cell hypoxia, oxidative stress, extracellular matrix remodeling, and angiogenesis/lymphogenesis 12. While CVD is a less serious condition, in prior work we also detected these changes in the placentas of women with venous insufficiency, indicating the possible impact of venous disease in these patients with CVD 9, 13-15. Chronic venous insufficiency has also been associated with a series of changes in cell signaling and in the behavior of smooth muscle cells. This determines a need to address the effects of this disease on the placenta in order to identify more effective therapeutic targets 16-18.

The metabolome has a large number of components that belong to a wide variety of compounds of different classes. These compounds are very diverse both in terms of their physical and chemical properties and in their concentration range. The surge and refinement of omics has allowed for the better characterization and knowledge of the expression profiles of the different metabolites in a large variety of placental pathologies and has also shown their potential use as biomarkers of these diseases 19-22. In this context, lipidomics is a growing area in the study of these metabolomic profiles. Lipids are the essential components of biological membranes and play multiple important roles in biological systems. Several studies have shown the differential expression of different groups of lipids in women with vascular hypertension 23, 24. This study accordingly sought to examine the lipid and metabolomic profiles of placental chorionic villi in women with CVD through ultra-high performance liquid chromatography coupled to mass spectroscopy (UHPLC-MS).

## Materials and Methods

### Experimental procedure

The appropriate study of a metabolic profile is based on the capacity to determine changes in biological fluid or tissue metabolites. However, there is no existing platform or method to examine the entire metabolome of a biological sample due to a wide concentration range of metabolites and their wide chemical diversity. The technique UHPLC-MS is adequate for this type of analysis because of its high sensitivity, wide coverage of metabolites, high performance, high throughput and wide dynamic range. In this study we used platforms based on UHPLC-MS to examine endogenous analytes 25-29.

### Tissue samples

Participants were informed of the study details and written consent was obtained from each woman in the third term of pregnancy visit. All candidates underwent examination using an Eco-Doppler 7.5 MHz transducer (M-Turbo Eco-Doppler, SonoSite, Inc.) to assess the venous system in the legs. The project received approval from the Committee on Clinical Research Ethics of the Hospital de Defensa Gómez-Ulla-UAH (37/17). Exclusion criteria were women with endocrine disorders such as diabetes mellitus, high arterial blood pressure; a body mass index (BMI) ≥ 25 kg / m²; non healthy habits; active infectious disease; autoimmune diseases; vein malformations; chronic renal disease, heart disease or lung disease; preeclampsia and/or hemolysis, elevated hepatic enzymes and low platelet syndrome; intrauterine growth restriction of known cause; pathological lesions such as placental infarction, avascular villi, late maturation and chronic inflammation affecting chorionic villi and the appearance of any of the exclusion criteria mentioned at any time point before delivery as well as prior evidence of CVD. Placentas were obtained immediately after childbirth and chorionic villus samples were obtained from 6 pregnant women with CVD classified according to CEAP criteria (C>1) 30 and 6 pregnant women without CVD used as healthy controls (HC). The mean age was 34.00±4.85 CVD and 32.33±7.11 HC.

### Sample preparation and metabolite extraction

Several UHPLC-MS platforms were used for wide coverage of the metabolome of chorionic villi 31-33. Metabolite extractions involved separating the samples into groups of species with similar physicochemical properties through the use of appropriate organic solvent combinations. Three platforms based on UHPLC-MS were employed to obtain an optimal profile of 1) fatty acyls, bile acids, steroids and lysoglycerophospholipids; 2) glycerollipids, glycerophospholipids, cholesteryl esters and sphingolipids; and, 3) amino acids.

Chorionic villi were washed in a 0.85 NaCl% solution to remove blood from tissue. Samples were enriched with chloroform (20:1 v/w) with a methanol solution containing metabolites not detected in the chorionic villi according to internal standards and prepared in platform 1 (40:1 v/w), and a 2:1 chloroform-methanol mixture containing the internal standards prepared in platform 2 (10:1 v/w). To homogenize the results of the mixture, a Precellys 24 homogenizer was used at 6500 rpm for 23 s. Once the samples had been incubated at -20 °C for 1 h and then centrifuged, the two different phases were collected:

#### Platform 1

Supernatants were collected following centrifugation at 18,000 × g for 15 min, dried, reconstituted in methanol, resuspended for 20 min and centrifuged (18,000 × g for 5 min at 4 °C) before their transfer to vials for UHPLC-MS.

#### Platform 2

Extracts were mixed with ammonium hydroxide in water (pH 9) and after brief centrifugation; the samples were incubated for 1 h at -20 °C. After their centrifugation at 18,000 × g for 15 min at 4 °C, the organic phase was collected. The dry extracts were reconstituted in acetonitrile:isopropanol (1:1), resuspended by shaking for 10 min, centrifuged (18,000 ° g for 5 min at 4 °C) and transferred to vials for UHPLC-MS analysis.

#### Platform 3

10 μl-aliquots of the extracts prepared for platform 1, were transferred to microtubes for subsequent amino acid analysis.

In addition, two types of quality control (QC) samples were set up to assess the quality of data obtained according to prior studies 34.

### UHPLC-MS analysis

A different UHPLC-MS method was used for each platform: chromatographic separations and the mass spectrometry detection conditions are detailed in **Table 1.** Stability was observed of the retention time (generally < 6 s of variation between one injection and the next), mass precision (normally < 3 ppm for m/z 400-1000 and < 1.2 mDa for m/z 50-400) and the system's sensitivity. The general quality of the analysis procedure was controlled for using repeat extracts of the QC sample for validation.

### Statistical analysis and interpretation of results

Data pre-processing generated a list of chromatography peak areas for the metabolites detected in each sample injection. A linear detection range was defined for each metabolite identified, assuming similar detector response levels for all metabolites belonging to a given chemical class, and represented by a single standard compound. Data points outside their corresponding linear detection range were replaced with missing values, and metabolites for which more than 30% of data points fell outside the corresponding linear range were excluded from data analysis.

**Figure 1:**
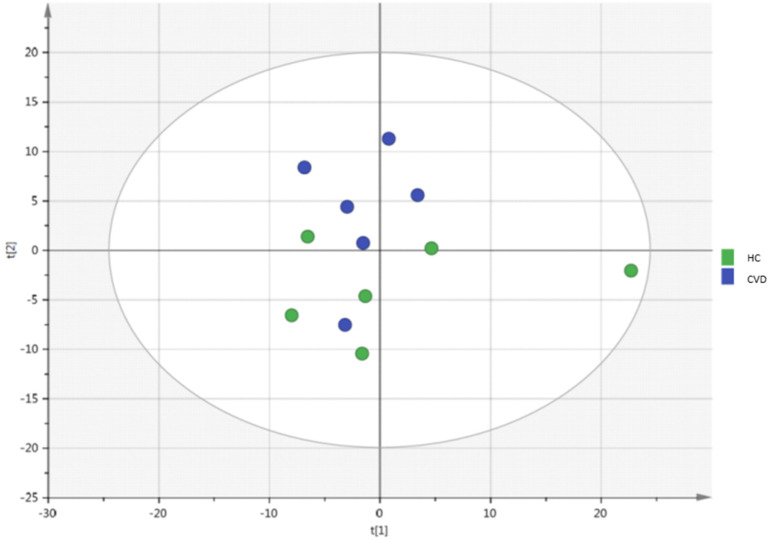
PCA analysis of chorionic villus samples from women with chronic venous disease (CVD) and healthy controls (HC). Model diagnostics: A = 2, R2X = 0.46, Q2X = -0.0172.

Data normalization was conducted according to the procedure described by Martínez -Arranz et al. 35. Once normalized, the dimensionality of the dataset was reduced to aid visualization of metabolic groupings in the different samples. This was conducted through multivariate data analysis including unsupervised principal components analysis (PCA) 36 and/or supervised orthogonal projections to latent structures (OPLS) analysis 37,38. Univariate statistical analyses were also run, calculating group percentage differences and unpaired Student's *t*-test *p* values (or Welch t-test when variances were unequal) for the groups CVD versus HC. Five samples were used to configure the extraction method, and different extractions performed on each sample. Great variability was observed among the different extractions of a single sample. As this variation was greater for certain metabolites, these were not considered and were thus withdrawn from the study. In total, 240 metabolic signatures were detected in the chorionic villus samples analyzed, which were included in the subsequent analysis of univariate and multivariate data. The benefits of using both univariate and multivariate approaches for data extraction have been recently reviewed 39. Both approaches are complementary and their results need not necessarily concur. Gross intensity results per metabolite and metabolic class are provided as supplementary material. Metabolic classes were calculated as the sum of normalized areas of all metabolites with the same chemical characteristics. The PCA plot for the villus and QC samples is shown in Figure 1S. The close proximity and overlapping of the QC injections indicate the good reproducibility and quality of measures. After validating the experiment's quality, QC injections were eliminated from the analysis. All data were processed using the TargetLynx application manager for MassLynx 4.1 software (Waters Corp., Milford, CT, USA).

## Results

### Pregnant women with CVD show elevated chorionic villus levels of lysophosphatidylcholines and amino acids

**Figure 2:**
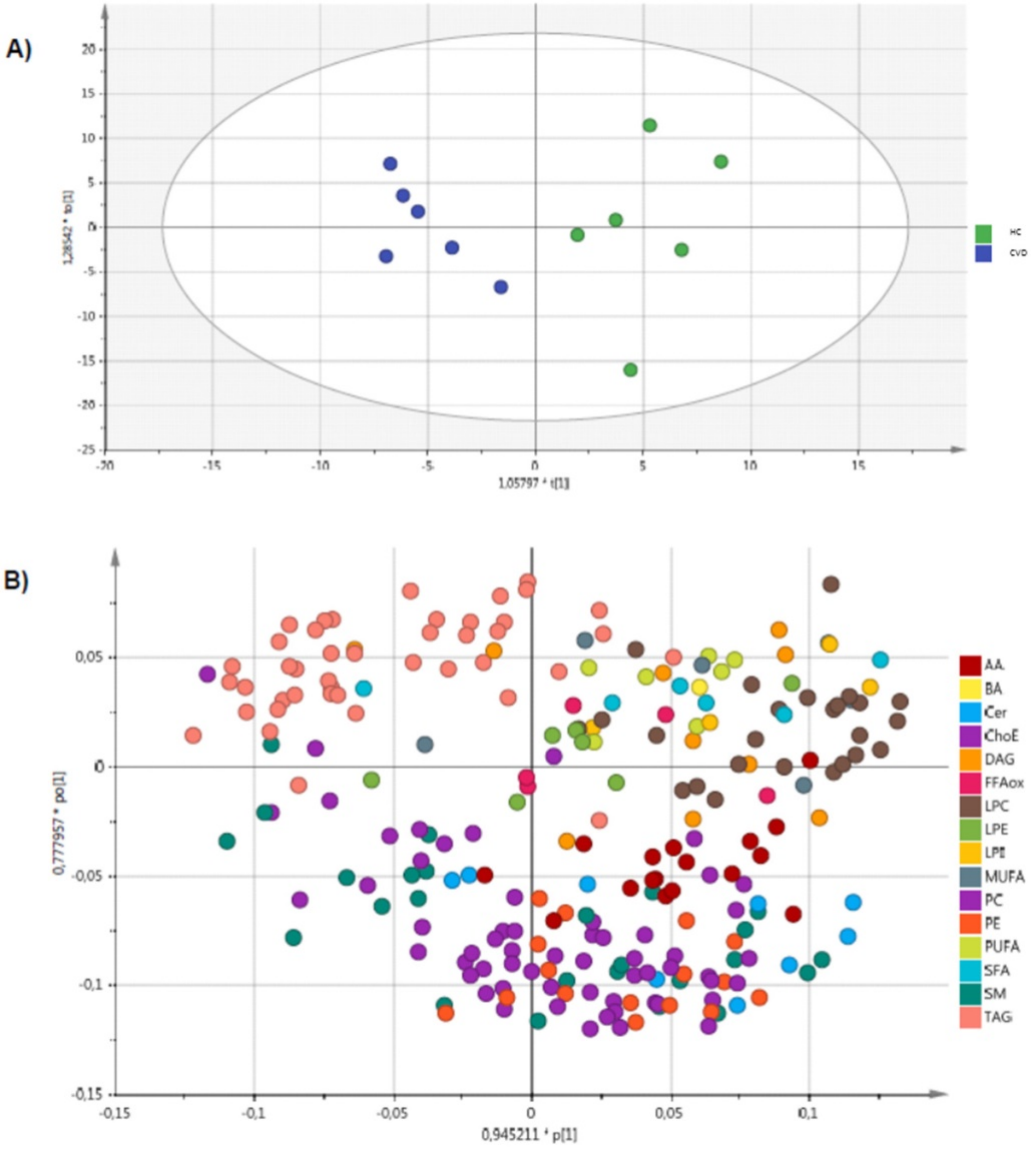
OPLS-DA analysis of chorionic villus samples from women with CVD and HC. Model diagnostics: A = 1 + 1 + 0, R2X = 0.357, Q2X = 0.251.

An unsupervised PCA model was generated through multivariate analysis with all the samples as shown in **Figure 1.** No clear separation was observed between the CVD and HC groups. A supervised OPLS-DA model was also constructed to achieve maximum separation between the two sample groups (**Figure 2**). The scatterplot (**Figure 2A**) shows the maximum separation observed between both groups. The metabolite species driving this pattern can be observed in the loading plot (**Figure 2B**). Metabolites found further from the plot origin (vertical zero axis) have a greater impact on the model while negatively correlated variables appear on opposite sides of the origin. While it can be inferred that lysophosphatidylcholines and amino acids are elevated in the placental chorionic villi of pregnant women with CVD, levels of triglycerides (TAG) and some sphingomyelins seem higher in the samples obtained in pregnant women without CVD. Notwithstanding, the model's predictive power was low (Q2X= 0.251).

### Pregnant women with CVD show significantly different chorionic villus levels of triacylglycerols compared to controls

**Figure 3:**
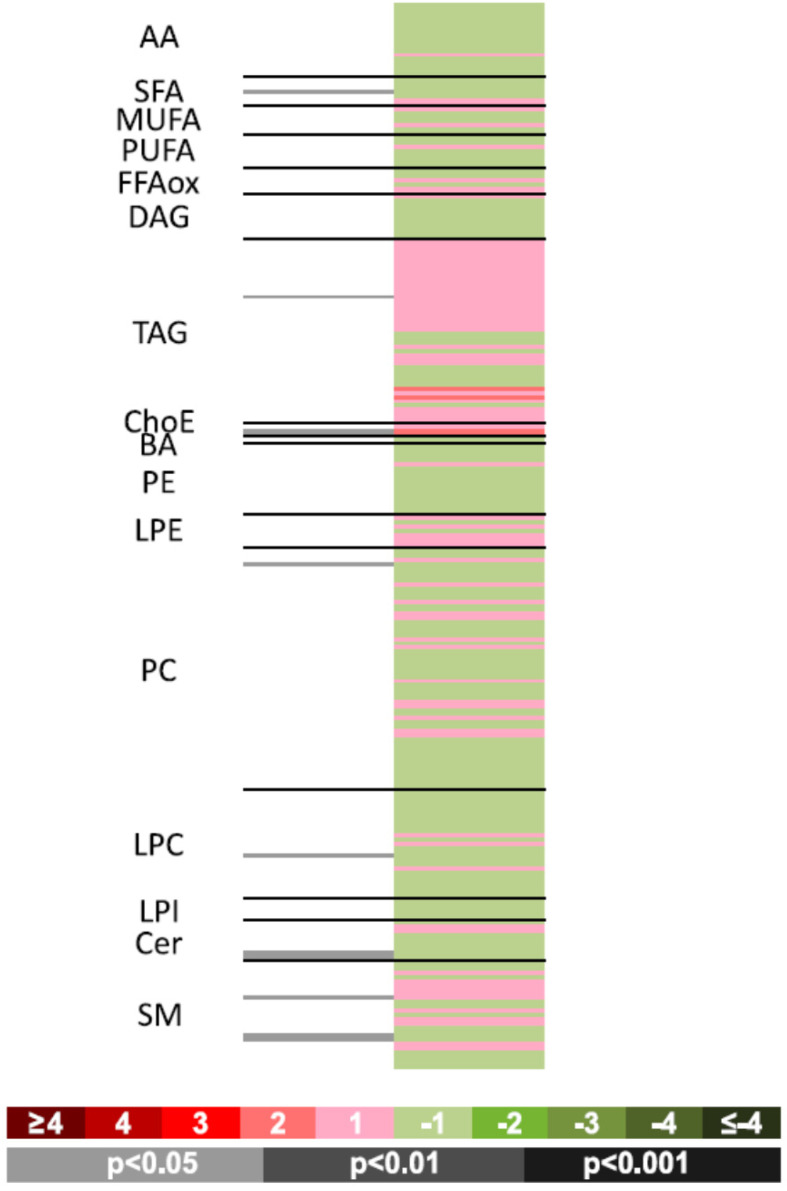
Heatmap showing individual metabolomic profiles obtained for the women with CVD vs. HC. Color codes for log2 (fold-changes) and *p* values obtained in paired Student t-tests are provided in the lower part of the figure.

To compare the two sample groups, univariate analyses were used to calculate percentage changes produced within the groups and the paired Student's *t* test (or Welch *t* test when variances were unequal) for intergroup comparisons. This information is provided as supplementary material. A heatmap was prepared to visualize comparisons (**Figure 3**). This figure shows fold-changes (log2) in the 240 metabolites examined along with the *p* values obtained in the Student *t* test for comparisons between the groups. Proportions of log-transformed ion abundance ratios are represented as a color-coded scale: the darker greens and reds indicate greater differences in metabolite levels between one group and the other. The gray lines indicate significant fold changes in individual metabolites, with the darker grays used to highlight the greater significance levels. It should be noted that metabolites are ordered in the heatmap according to the number of carbons and degree of unsaturation of their acyl chains. This heatmap offers easy visualization of differences between chorionic villus samples obtained from pregnant women with CVD and HC.

**Figure 4:**
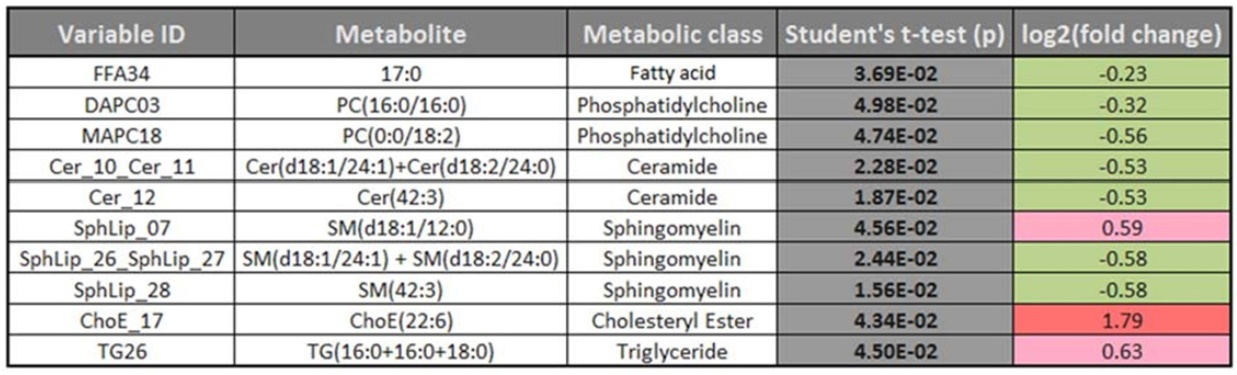

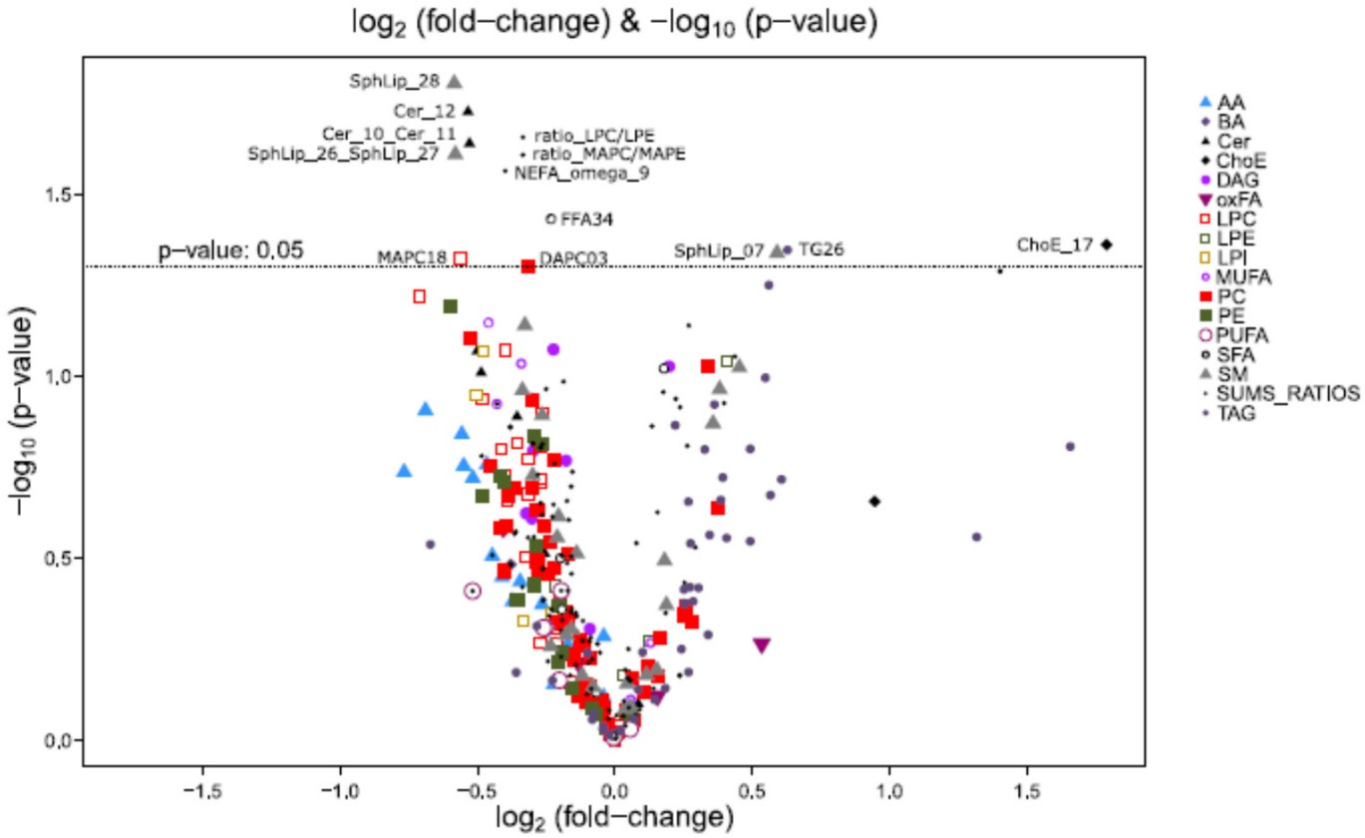
Vocano plots [-log10 (p value) vs. log2 (fold-changes)] for the comparison between women with CVD vs. HC.

The complete metabolomic profile of the chorionic villi of the women with CVD seemed modified in relation to the HC profile. Only TAG levels were higher in the CVD cases. Despite the small sample size of this preliminary study, significant differences between cases and controls should be highlighted such as in some sphingolipids, ceramides (Cer) and other lipid species described below. To complement the heatmap results, we prepared a volcano plot in which significance [-log10 (p value) is plotted against log2 change magnitude (*fold change*)] to compare the CVD and HC groups (**Figure 4**). This plot identifies the individual metabolites varying significantly between the two groups. Accordingly, **Table 2** details the metabolites showing significant differences (*p* < 0.05). The significantly higher levels of the sphingolipid SphLip_07 (SM (d18: 1/12: 0)) in the chorionic villi of women with CVD should be highlighted.

### Pregnant women with CVD show significantly different chorionic villus levels of omega 9 non-esterified fatty acids compared to controls

Besides considering individual metabolites, the volcano plot also provides results of lipid classes and proportions. Lipid classes were calculated as the sum of normalized areas of all metabolites with similar chemical characteristics. The substrate product ratio of an enzyme provides an empirical estimate of reaction velocity. These proportions were calculated to understand possible enzyme activities related to lipid metabolism. These data give an idea of the directions of chemical reactions. The only class of lipids emerging as modified in the CVD sample group was that of omega 9 non-esterified fatty acids compared to the HC sample group (*p* = 0.027). In addition, we observed a lower ratio of monoacylglycerophosphocholine to monoacylglycerophosphoethanolamine (MAPC/MAPE) in the CVD group (*p* = 0.025).

## Discussion

CVD is highly prevalent in women during pregnancy. As interest mounts in linking this condition to different placental damage indicators whose elevated levels could compromise maternal-fetal well-being 40, it is essential that we continue exploring possible homeostatic alterations produced in this disease along with their repercussions on the placenta. The findings of our study indicate the presence of markers of increased lipid peroxidation in patients with CVD, suggesting the possible impacts of this condition on lipid components 41. This study is the first to detect a difference in the lipid profiles of pregnant women with CVD in relation to healthy pregnant women. We observed variations in a great variety of lipids, spanning from fatty acids to complex lipids. Such alterations could have important repercussions on placental function and their involvement in different complications of pregnancy has been also described 42.

Membrane lipids were especially affected by CVD. We detected abnormal levels of some of these molecules such as phosphatidylcholines or sphingomyelins. It should be underscored that these cell membrane components carry out important functions in gestation and occur in different proportions in different tissues 43, 44. Decreased sphingomyelin levels have been linked to pathological conditions such as maternal malperfusion 45. Here, we noted diminished levels of these lipids in women with CVD, suggesting possible implications of these molecules for assessing the state of the placenta and maternal-fetal well-being. Ceramides are lipid molecules synthesized from sphingomyelin molecules as the result of the actions of sphingomyelinase 46. These substances are known to play a major role in regulating the cell response to different stress conditions by controlling key processes such as apoptosis or the cell cycle 47. Our results indicate a significant reduction in these lipids, probably associated with diminished sphingomyelin levels. Such a loss in the placenta of an important regulating factor for tissue homeostasis could promote damage to this structure. Interestingly, the buildup of ceramides has been observed in preeclampsia and has been associated with increased autophagy of the trophoblasts that accompany this disease 48. In our study, one sphingomyelin, namely sphingomyelin SM (d18:1/12:0), rather than being diminished was found to be elevated in the pregnant women with CVD. According to De Guzman et al. 49, this molecule is a marker of tissue ageing and is reduced in conditions of restricted calorie intake. Our study suggests that the increase in this sphingomyelin could indicate the faster ageing of placental tissue in women with CVD.

Phospholipids are essential components of placental cell membranes especially needed for the exchange of molecules between the cell environment and its interior 50. Alterations in this function have been associated with some complications of pregnancy such as restricted intrauterine growth 51 or with the placental dysfunction observed in some women undergoing *in vitro* fertilization 52. In these patients, it has been proposed that increased levels of phosphatidylcholine and other membrane phospholipids could play an important role in placental dysfunction. The increased amount of phosphatidylcholine observed here in the placentas of women with CVD could indicate alterations in the transport of substances with the consequence of compromised tissue homeostasis in these patients.

Similarly, we were able to detect a significant increase in cholesteryl esters ChoE (22:6) in patients with gestational CVD. During pregnancy, cholesterol plays a major role as it is needed for maternal-fetal interchange of substances or for the synthesis of important metabolic products including placental progesterone and estrogen 53. Excess cholesterol builds up as cholesteryl esters in the form of lipid droplets within the cell 54. Thus, the increase in ChoE observed could indicate their greater deposition. Similarly, we also detected increased levels of TAG in participants with CVD compared to controls. Our results are consistent with those of Brown et al. 55 who observed abnormal lipid deposits in the placentas of women with preeclampsia, adding to the evidence of similar pathogenic mechanisms of the two conditions.

Finally, our study revealed a reduction in the unsterified omega 9 fatty acid found mainly in olive oil. Like other unsaturated fatty acids, omega 9 plays an important role in regulation of the inflammatory response and in the prevention of several diseases 56, 57. Diminished levels of this acid could be related to the modified capacity of the immune system to control inflammation in the placenta, which could induce placental tissue damage. We also noted an alteration in the MAPC/MAPE ratio suggesting the modified activity of lysophosphatase in pregnant women with CVD.

## Conclusions

Our findings reveal subtle differences in the lipid expression profiles of women with and without CVD during pregnancy. As some of these metabolite alterations are common to other more severe vascular diseases such as preeclampsia, there is a need for further work in this area. These results provide direction for future research efforts targeted at identifying the lipid modifications produced in the placentas of women with CVD along with the pathogenic mechanisms that could be underlying these changes and their possible effects on maternal-fetal well-being.

## Supplementary Material

Supplementary tables.

## Figures and Tables

**Table 1:** UPLC®-MS procedures and conditions used for each platform

	Platform 1	Platform 2	Platform 3
Column type	UPLC BEH C18, 1.0 × 100 mm, 1.7 μm	UPLC BEH C18, 2.1 × 100 mm, 1.7 μm	UPLC BEH C18, 1.0 × 100 mm, 1.7 μm
Flow rate	0.14 ml/min	0.40 ml/min	0.14 ml/min
Solvent A	H2O + 0.05% Formic Acid	H2O + ACN + 10 mM Ammonium Formate	10 mM Ammonium Bicarbonate (pH= 8.8)
Solvent B	ACN + 0.05% Formic Acid	ACN+ Isopropanol + 10mM Ammonium Formate	ACN
(%B), time	0%, 0 min	40%, 0 min	2%, 0 min
(%B), time	50%, 2 min	100%, 10 min	8%, 6.5 min
(%B), time	100%, 13 min	40%, 15 min	20%, 10 min
(%B), time	0%, 18 min	40%, 17 min	30%, 11 min
(%B), time	-	-	100%, 12 min
(%B), time	-	-	2%, 14 min
Column temperature	40 °C	60 °C	40 °C
Injection volume	2 μl	3 μl	2 μl
Source temperature	120 °C	120 °C	120 °C
Nebulisation N2 flow	600 l/hour	1000 l/hour	600 l/hour
Nebulisation N2 temperature	350 °C	500 °C	350 °C
Cone N2 flow	30 l/hour	30 l/hour	10 l/hour
Capillary voltage	2.8 kV	3.2 kV	3.2 kV
Cone voltage	50 V	30 V	30 V

**Table 2:** Significant differences (*p* <0.05) in individual metabolites for the women with CVD vs. HC. Color codes for log2 (fold-changes) and *p* values as in the heatmap in Fig. 3.

## Abbreviations

AA: amino acids
AC: acyl carnitines
ArAA: aromatic amino acids
BA: bile acids
BCAA: branched chain amino acids
Cer: ceramides
ChoE: cholesteryl esters
CMH: monohexosylceramides (cerebrosides)
DG: diacylglycerides
FAA: fatty acid amides (primary fatty amides)
FSB: free sphingoid bases
LPC: lysophosphatidylcholines
MAG: monoacylglycerides
MUFA: monounsaturated fatty acids
NAE: N-acyl ethanolamines
NEFA: non-esterified fatty acids
oxFA: oxidized fatty acids
OPLS: orthogonal projections to latent structures
PC: phosphatidylcholines
PCA: principal component analysis
PE: phosphatidylethanolamines
PUFA: polyunsaturated fatty acids
SFA: saturated fatty acids
SM: sphingomyelins
